# Phenolic Profiles and DPPH Radical-Scavenging Activity of Two *Eranthis* Species from Southeastern Siberia

**DOI:** 10.3390/ijms27219973

**Published:** 2026-11-06

**Authors:** Vera A. Kostikova, Natalia V. Petrova, Alexander A. Chernonosov, Vladimir V. Koval, Maria V. Lyapunova, Yulia D. Razgulyaeva, Evgeniia R. Lazareva, Alexander A. Kuznetsov, Kun-Li Xiang, Wei Wang, Andrey S. Erst

**Affiliations:** 1 Central Siberian Botanical Garden, Siberian Branch of Russian Academy of Sciences (SB RAS), Novosibirsk 630090, Russia; 2 Komarov Botanical Institute of Russian Academy of Sciences, St. Petersburg 197022, Russia; npetrova@binran.ru; 3 Institute of Chemical Biology and Fundamental Medicine, Siberian Branch of Russian Academy of Sciences (SB RAS), Novosibirsk 630090, Russia; alexander.chernonosov@niboch.nsc.ru (A.A.C.); koval@niboch.nsc.ru (V.V.K.); 4 Department of Natural Sciences, Novosibirsk State University, Novosibirsk 630090, Russia; erlazareva@frcftm.ru; 5 Faculty of Chemistry, National Research Tomsk State University, Tomsk 634028, Russia; lyapunova.mari@mail.ru (M.V.L.); yrazgulyaeva@yandex.ru (Y.D.R.); 6 Laboratory of the Herbarium, Tomsk State University, Tomsk 634050, Russia; ys.tsu@mail.ru; 7 State Key Laboratory of Plant Diversity and Specialty Crops, Institute of Botany, Chinese Academy of Sciences, Beijing 100093, China; kunlixiang@ibcas.ac.cn (K.-L.X.); wangwei1127@ibcas.ac.cn (W.W.)

**Keywords:** *Ranunculaceae*, *Eranthis sibirica*, *Eranthis tanhoensis*, phenolic compounds, antioxidant activity

## Abstract

This study focused on *Eranthis sibirica* and *E. tanhoensis* (Ranunculaceae), endemic species of Southeastern Siberia. Until recently, these plants had not been sufficiently studied from a phytochemical perspective. The aim of this work was to analyze the phenolic composition and antioxidant properties of 70% ethanolic extracts of their leaves. Liquid chromatography coupled with high-resolution mass spectrometry (LC-HRMS) was used to analyze metabolites in *Eranthis sibirica* and *E. tanhoensis* extracts. The free-radical-scavenging ability of the extracts was assessed using the DPPH method. LC–HRMS metabolomic profiling of the leaf extracts from *E. sibirica* and *E. tanhoensis* revealed a diverse range of phenolic constituents; among them, 25 compounds were annotated for the first time for this genus, while the remaining 19 had been previously reported. These included flavonols (16 components), flavanones (8), flavones and isoflavones (5), chalcones (5), flavans (1), catechins (1), and phenolic acids and their derivatives (8). The *E. tanhoensis* leaf extracts were found to have higher phenolic content (total phenolic content of 3.07 g per 100 g dry weight) and greater DPPH radical scavenging activity (IC_50_ = 268.45 μg/mL dry weight) than *E. sibirica*. These findings indicate that the species with the higher phenolic accumulation also displays stronger antioxidant activity, suggesting that both species—particularly *E. tanhoensis*—warrant further phytochemical and biological investigation.

## 1. Introduction

Phenolic compounds constitute the most extensive class of secondary plant metabolites. Due to the structural characteristics of their carbon skeleton and the diversity of functional groups, they are categorized into numerous classes, such as phenolic acids, flavonoids, tannins, and others. In animal organisms, phenolic structures serve as the basis for several neurotransmitters (e.g., dopamine and norepinephrine) and other bioactive substances. In addition to their broad capacity to neutralize free radicals, representatives of this class possess a diverse spectrum of therapeutic properties: from bactericidal and fungicidal activities [1,2] to cardioprotective, neuroprotective, and hepatoprotective effects [3,4]. Furthermore, they demonstrate immunomodulatory and anti-inflammatory properties [5,6] and effectively inhibit mutagenesis and tumor metastasis [7].

A particular focus must be placed on the study of these secondary metabolites in terms of their antioxidant activity: the ability to bind various types of free radicals as well as other highly reactive oxygen and nitrogen compounds that cause oxidative stress. These compounds play a pivotal role in preventing and treating a wide range of pathologies, from malignancies to age-related diseases [8,9]. Diverse members of the Ranunculaceae family represent a promising source for the discovery of potent phenolic compounds.

Some species of the family Ranunculaceae are used in folk and traditional medicine (*Thalictrum foetidum* L., *Adonis amurensis* Regel & Radde, etc.), while others are considered toxic (*Aconitum soongaricum* Stapf) [10]. The majority of the genera are currently understudied. These include the genus *Eranthis* L., which comprises early spring ephemeroid perennials. Species of the genus *Eranthis* are of particular interest for research due to their disjunct distribution. Plant species of the section Shibateranthis (Nakai) Tamura are distributed in the temperate zone in the east, from northern and eastern Asia and the Himalayan region of China to Japan. Plants of the section Eranthis are distributed in America, Europe, and southwestern and central Asia [11]. Secondary metabolites of *Eranthis* species have been poorly investigated. The presence of chromones, triterpene saponins, flavonoids, etc., in these plants has been confirmed [12]. However, the broad spectrum of biological activity identified in certain species and the diversity of biologically active compounds (Erst et al., 2023) make this genus a promising candidate for further research [12].

*Eranthis tanhoensis* and *E. sibirica* are two morphologically similar species that grow in the same climate zone in the mixed forests of southeastern Siberia. They bloom in May and reach full maturity in July [11].

Studies are currently underway to identify the optimal conditions for the production and culturing of *E. tanhoensis* callus tissue. This opens up opportunities for the production of biologically active substances for the cosmetic and pharmaceutical industries [12]. Data on the content of biologically active compounds in *E. tanhoensis* and *E. sibirica* are very limited. Flavonoids (quercetin and kaempferol) were identified in the leaves of *E. tanhoensis*, and vitexin and orientin were found in the leaves of *E. sibirica*. In addition, chlorogenic, caffeic and salicylic acids were found in both species [12]. Although many polar organic solvents (methanol, acetone, and ethyl acetate) are used for the extraction of phenolic compounds, these studies employed the 70% (*v*/*v*) aqueous ethanol leaf extracts of *E. tanhoensis* and *E. sibirica*. This choice is due to the combination of ethanol and water as an environmentally benign and biocompatible solvent for preparing extracts intended for oral pharmaceutical dosage forms.

The presence of phenolic compounds in *E. sibirica* and *E. tanhoensis* has been previously reported; however, the data remain limited. The present study was specifically designed to provide a comprehensive comparison of these two species to extend the existing data. Its novelty lies in a more extensive, detailed LC-HRMS profiling of their phenolic constituents, coupled with a comparative evaluation of their antioxidant activity via the DPPH assay. Thus, we aimed to systematically characterize the phenolic composition of the leaf extracts and compare the antioxidant potential of these two Siberian plant species.

## 2. Results

### 2.1. Antioxidant Activity Assay Using 2,2-Diphenyl-1-Picrylhydrazyl (DPPH) E. tanhoensis and E. sibirica Leaf Extracts

This study reports, for the first time, the antioxidant activity of the 70% (*v*/*v*) aqueous ethanol leaf extracts of *E. sibirica* and *E. tanhoensis* collected from natural populations on the southwestern shore of Lake Baikal. Antioxidant activity was determined using the DPPH radical-scavenging assay. The results were expressed as IC_50_ values (Table 1). The *E. tanhoensis* leaf extract is more active than *E. sibirica*, but much less potent than the reference antioxidants.

### 2.2. Phenolic Antioxidant Content

The contents of the total flavonoids (including catechins), phenolic acids, tannins, and total phenolic compounds were calculated. All investigated phenolic classes showed significantly higher concentrations in *E. tanhoensis* extracts (Table 1).

### 2.3. Phenolic Compound Content in E. tanhoensis and E. sibirica Leaves

The LC-chromatograms of the *E. tanhoensis* and *E. sibirica* leaf extracts were obtained (Figure S1). The LC-HRMS analysis revealed more than 200 components, of which 44 compounds were confirmed or annotated as phenolic substances. The study showed that both extracts contain an identical set of phenolic compounds (Table 2). The detected components were classified as flavonols (16 components), flavanones (8 components), flavones and isoflavones (5 components), chalcones (5 components), flavan (1 component), and catechins (1 component). The compounds were identified using reference standards or annotated with international databases, including mzCloud, mzVault, and ChemSpider.

## 3. Discussion

### 3.1. Antioxidant Activity of E. tanhoensis and E. sibirica Leaf Extracts and Their Content of Phenolic Antioxidants

The leaf extract of *E. tanhoensis* (IC_50_ = 268.45 μg/mL) exhibited 1.5-fold higher antioxidant activity against the DPPH radical compared to the *E. sibirica* leaf extract (IC_50_ = 416 μg/mL). The *E. tanhoensis* leaf extract also shows relatively high antioxidant activity compared to other previously studied plants. For example, similar methods were used to investigate the antioxidant activity of seeds, leaves, and stems of *Silybum marianum* L. (milk thistle), which has potential applications in medicine, cosmetology, and nutrition, including as a source of antioxidants [16]. Milk thistle enhances antioxidant activity, improves nutritional value, and supports the viability of yogurt starter cultures, contributing to the stability and shelf life of functional yogurt [17]. The *S. marianum* leaf extract demonstrated the highest antioxidant activity in DPPH assays (IC_50_ = 388.16 μg/mL, with an ascorbic acid value of 81 μg/mL). In contrast, the seeds did not show significant free-radical-scavenging activity. The authors of the study attribute the difference in antioxidant activity between the leaves and seeds to variations in the chemical composition of extracts from different plant parts, as not all antioxidants can react with DPPH. Some antioxidants may possess specific reactivity toward other radicals or exert their antioxidant activity through different mechanisms, which are not captured by the DPPH assay [16]. In the Phytochemistry Laboratory of the CSBG, using the same equipment as in the present study and the same reference values obtained for Trolox and ascorbic acid, IC_50_ values of 38.3 μg/mL and 102.1 μg/mL were obtained for dry extracts from the aerial parts of *Spiraea salicifolia* L. and *Spiraea media* Schmidt., respectively. Plants of the genus *Spiraea* are traditionally recognized as a valuable source of antioxidants [18].

In addition to higher antioxidant activity, a higher content of potential antioxidant compounds was identified in *E. tanhoensis* compared to *E. sibirica*. The *E. tanhoensis* leaf extract has 3-fold higher catechin content, 2.9-fold higher flavonoid content, 1.6-fold higher phenolic acid content, 1.4-fold higher phenolic compound content, and 1.2-fold higher tannin content (Table 1). Differences in the concentration of the studied phenolic substances may be due to several factors: species membership, environmental conditions, developmental stage, or analytical methodology [19,20]. In this study, we controlled for some of these variables: the investigated species were at the fruiting stage, occurred within the same climatic zone at similar altitudes, and were subjected to identical sample preparation and investigation procedures. High levels of phenolic compounds may be associated with both genetic characteristics and specific plant responses to external stress, such as injury or infection [21]. Therefore, we recognize certain limitations of this study. Further research is required to reliably identify the factors influencing the difference in the amount of active compounds in *E. sibirica* and *E. tanhoensis*. Recent data show that *E. sibirica* leaves possess a greater diversity of phenolic compounds compared to *E. tanhoensis* [11]. The higher antioxidant activity of *E. tanhoensis* leaves may be attributed to an elevated content of specific phenolic compounds with potent free-radical-scavenging properties.

These findings underscore the potential of the genus *Eranthis* as a significant source of novel natural antioxidants, warranting more detailed phytochemical investigations to isolate and characterize specific bioactive constituents.

### 3.2. Phenolics of E. tanhoensis and E. sibirica Leaf Extracts Detected by LC-HRMS

Data on the flavonoid content in species of the genus *Eranthis* are meager [12], yet flavonols are characteristic of this genus. Previous studies reported the presence of flavonols in *E. hiemalis* [22], *E. stellata* and *E. tanhoensis* [13], and *E. longistipitata* Regel. [14]. The materials obtained in the studies support this assumption: quercetin (**1**), kaempferol (**11**), and their derivatives are most widely represented. The diversity of flavonols in the studied species is due to the addition of one (rarely two or three) sugar residues, mainly at the C-3 position. Sugar components are represented by various combinations of glucose, rhamnose, xylose, and arabinose residues. The majority of the detected flavones are O-glycosides, although C-glycosides are also present in the *E. tanhoensis* and *E. sibirica* extracts. These compounds include orientin (**26**) and carlinoside (**27**). Carlinoside (**27**) was previously identified in *E. longistipitata* [14], and orientin (**26**) was found in *E. sibirica* and *E. stellata* Maxim. [13]. Among flavones, tentatively annotated norwogonin (**25**) or 5,7,8-trihydroxyflavone is of particular interest. This compound was previously described only in species of the genus *Scutellaria* L. [23,24]. Norwogonin (**25**) is a pharmacologically active flavone [25]; however, its content in plants is low, and it is mainly accumulated in the subterranean part [26]. Flavanones, often referred to as dihydroflavones, are compounds that lack a double bond between the C-2 and C-3 atoms in the C-ring of the flavonoid skeleton. Previously, flavanones (e.g., 6-methoxytaxifolin) were found only in *E. longistipitata* [14]. In this study, *E. tanhoensis* and *E. sibirica* exhibited a number of compounds formed through the addition of three hydroxyls and an additional methyl group to the basic flavanone molecule (as in 5,7,4’-trihydroxy-6-methylflavanone) (**31**), or a sugar moiety (as in prunin (**34**)), etc. Chalcones (**19**–**22**) also attract considerable attention because they contain a ketoethylenic moiety and a reactive α,β-unsaturated carbonyl group and are promising candidates for drug development. Due to their unique structure, chalcones can serve as starting compounds for synthesis of a variety of new heterocyclic drugs with superior biological activity compared to existing standards [27,28].

Non-flavonoid phenolic components were phenolic acids and their derivatives (**37–44**). In this study, eight components were detected. The most frequent were derivatives of benzoic acid, a phenolic compound with a carboxyl group attached to the aromatic ring, present in the tissues of most plants. In addition to benzoic acid (**37**), gentisic acid (**38**), 4-(octyloxy)benzoic acid (**39**), 4-isobutylbenzoic acid (**40**), and methyl salicylate (**41**) were detected in the extracts of the studied *Eranthis* species. Derivatives of cinnamic acid such as O-coumaric acid (**42**), caffeic acid (**43**) and the natural diester of glucose and p-coumaric acid, 1,6-Bis-O-[(2E)-3-(4-hydroxyphenyl)-2-propenoyl]-β-D-glucopyranose (**44**), were also found in the extracts. Previous studies reported quantitative data on the content of salicylic and caffeic acids in *E. tanhoensis* and *E. sibirica* [13]. Other phenolic acids have been reported for these species for the first time.

It should be noted, however, that we focus on characterizing the chemical diversity rather than the quantitative abundance of the metabolites, as we recognize that LC-HRMS annotation alone is insufficient to prove that these compounds are present at biologically meaningful concentrations.

Phenolic compounds are secondary metabolites widely distributed in higher plants. Wound stress in plants triggers synthesis of phenolic compounds as a resistance mechanism for healing and repair. These secondary metabolites can powerfully neutralize reactive oxygen species, which are widely used in pharmaceuticals [29]. In addition, plant phenolic compounds have been found to inhibit lipid peroxidation in humans, which has pharmaceutical value [30].

Many of the phenolic secondary metabolites identified in the leaves of *E. tanhoensis* and *E. sibirica* possess documented antioxidant properties. Relevant literature data are summarized in Table 3. While this table serves as a valuable reference, it should not be used to quantitatively deduce which specific compounds drive the overall activity of the extracts. This limitation arises because individual component concentrations were not quantified in this study, and the literature-derived IC_50_ values were obtained under varying experimental conditions. Furthermore, the total extract activity may be governed by complex additive, synergistic, or antagonistic interactions among its constituents.

Among the methods available for assessing antioxidant activity, the DPPH assay was selected for its operational simplicity, well-defined reaction mechanism, and solvent compatibility [31]. The radical scavenging efficiency of these compounds is closely linked to their chemical structures, particularly the OH groups, as outlined in the table. Notably, quercetin and kaempferol represent the most abundant and extensively studied flavonols in this context [44]. In plants, quercetin predominantly occurs as an aglycone or as glycosides, typically bound at the C-3 position [45]. Although such glycosides exhibit varying degrees of antioxidant activity [36,46,47], any assumption that the pronounced antioxidant potential of *E. tanhoensis* leaves stems from an elevated content of these specific phenolics remains strictly hypothetical and requires further quantitative validation.

## 4. Materials and Methods

### 4.1. Plant Material

**Figure 1:**
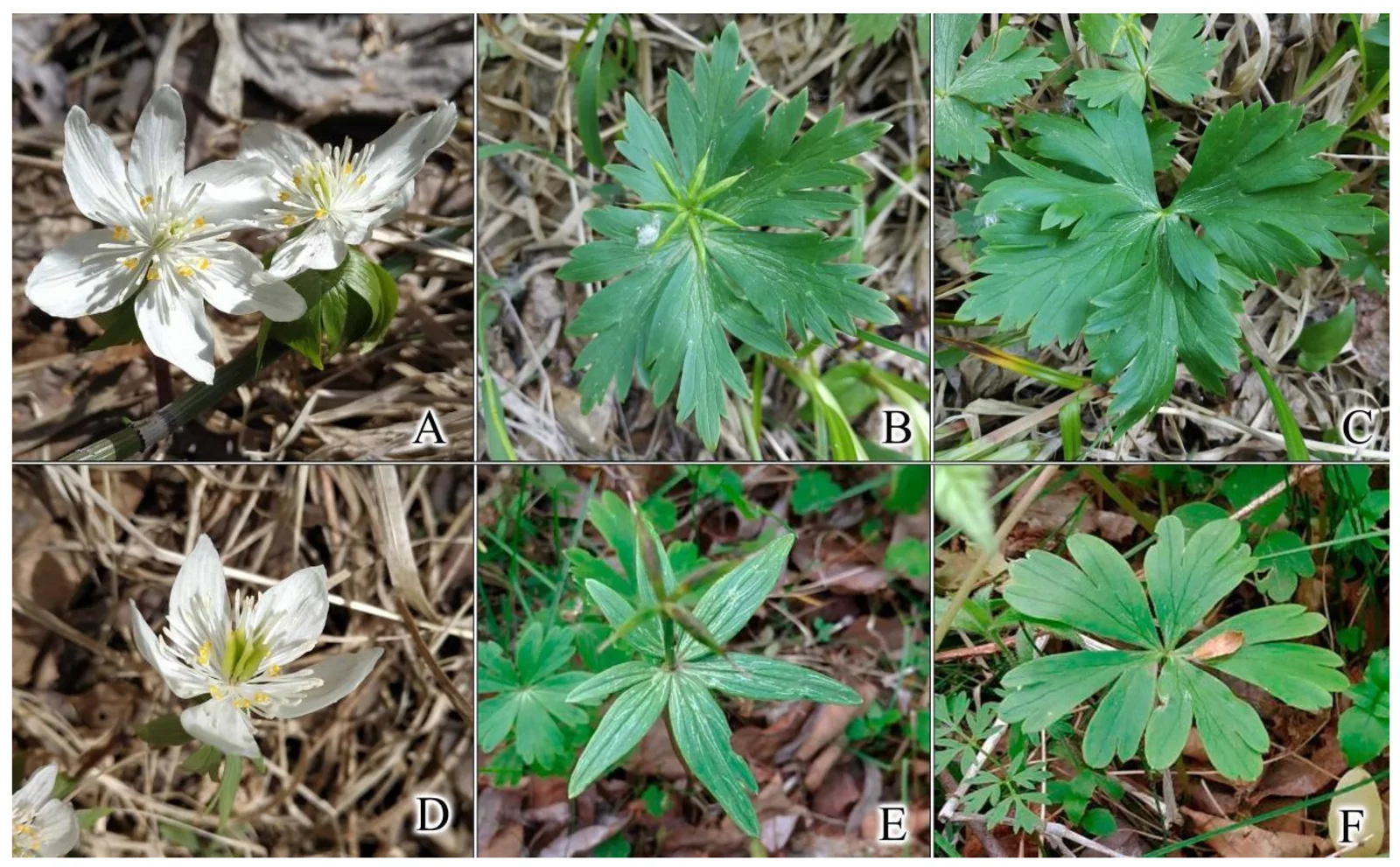
Morphological differences between *E. tanhoensis* (**A**–**C**) and *E. sibirica* (**D**–**F**): (**A**,**D**)—flowers; (**B**,**E**)—cauline leaves; (**C**,**F**)—basal leaves. Photo by Andrey S. Erst.

Leaves of *E. sibirica* and *E. tanhoensis* were collected by A.S. Erst and a team of researchers on 20–21 June 2019, in the area of the southwestern shore of Lake Baikal: Irkutsk region, Slyudyanka district, in the environs of the town of Slyudyanka, 531 m a.s.l. (*E. sibirica*; voucher specimen NS-0000956); Republic of Buryatia, Kabansky district, Tolbazikha River bank, 571 m a.s.l. (*E. tanhoensis*; voucher specimen NS-0000957). *Eranthis sibirica* differs from *E. tanhoensis* in lanceolate or oblanceolate segments of cauline (involucral) leaves (vs. rounded, rhombic), petals narrowly urn-shaped, margins between abaxial and adaxial lips white (vs. petals broadly urn-shaped, margins between abaxial and adaxial lips yellow) (Figure 1) [11]. The plants were collected and identified (based on the phenotype and morphological traits) by A.S. Erst, Ph.D., a Ranunculaceae specialist and Leading Researcher at the Central Siberian Botanical Garden, SB RAS. Plant leaves were collected from 20 to 30 specimens from one population. Leaf samples from each population were randomly divided into three parts. Each portion of leaves was ground, mixed, and subsequently subjected to independent extraction. Both species were in the fruiting stage and grew in the same climatic zone at similar altitudes, which makes them suitable for comparison. A key limitation of this study is that the data were obtained for species from a single sampling population and site. Consequently, the observed patterns may reflect local adaptations or site-specific environmental conditions rather than species-wide traits. Expanding the sampling effort to include multiple populations per species remains an important direction for future work.

### 4.2. Ethanol Extract Preparation

For the analysis of the composition and content of phenolic compounds, leaves of *E. sibirica* and *E. tanhoensis* were air-dried in shaded areas, crushed and sieved through a 2 mm sieve. After that, a representative sample was taken from the sieved material. An accurately weighed sample (300 mg) was extracted twice with 70% ethanol in a water bath at 80 °C under reflux: first with 30 mL for 30 min, then with 20 mL for 20 min. After filtration, the residue in the flask and on the filter was washed with 5 mL of 70% ethanol. Each of the obtained extracts had a total volume of 46 mL. The combined extract was used to analyze the content of biologically active compounds and to measure the antioxidant activity using the DPPH method. Three independent biological replicates were performed for each plant species.

### 4.3. Analysis of the Content of Phenolic Compounds

The content of phenolic compounds was determined spectrophotometrically using standard methods based on the change in the absorbance of the solution when interacting with specific substances.

The total phenolic content was determined using the Folin–Ciocalteu reagent [48]. Absorbance was measured at 765 nm using an SF-56 spectrophotometer (Lomo, St. Petersburg, Russia). The standard curve was constructed with gallic acid (concentration 0.002–0.010 mg/mL). The result was expressed as g gallic acid equivalent per 100 g of dry weight.

Flavonol content was determined spectrophotometrically based on the reaction of complex formation between flavonols and aluminum chloride [49]. Absorbance of the solution with aluminum chloride was measured using the SF-56 spectrophotometer (Lomo, St. Petersburg, Russia) at 415 nm in a 1 cm path-length cuvette, with acetic acid solution as the control. The level of flavonols was calculated from a rutin (Chemapol) calibration curve at concentrations of 0.002–0.010 mg/mL. The result was expressed as g rutin equivalent per 100 g of dry weight.

Catechin content was determined spectrophotometrically by the method based on the ability of catechins to produce crimson coloration in a solution of vanillin in concentrated hydrochloric acid [50]. Color intensity was measured using the SF-56 spectrophotometer (Lomo, St. Petersburg, Russia) at 504 nm in a 1 cm path-length cuvette. The standard curve was constructed with (±)-catechin (Sigma) at concentrations of 0.001–0.020 mg/mL. The result was expressed as g catechin equivalent per 100 g of dry weight.

Hydrolyzable tannins were quantified using the method proposed by L.M. Fedoseeva [51]. The intensity of the resulting color was measured at 420 nm using the SF-56 spectrophotometer (Lomo, St. Petersburg, Russia) and a 1 cm path-length cuvette. A government-standard sample of tannin (Sigma, concentration 0.002–0.010 mg/mL) served as a standard. The result was expressed as g tannin equivalent per 100 g of dry weight.

The total content of phenolic acids was determined using Arnov’s reagent [52]. Absorbance was measured immediately at 490 nm using the SF-56 spectrophotometer (Lomo, St. Petersburg, Russia). The standard curve was constructed with caffeic acid (Serva, concentration 0.02–0.10 μg/mL). The result was expressed as g caffeic acid equivalent per 100 g of dry weight.

A detailed description of the techniques for studying biologically active compounds is given in [53].

### 4.4. Antioxidant Activity Assay Using 2,2-Diphenyl-1-Picrylhydrazyl (DPPH)

The free-radical-scavenging ability of the extracts was assessed using the DPPH method [52,54]. For this purpose, the extracts were diluted with 70% ethanol to concentrations in the range of 20–1200 μg/mL. Next, a 2 mL aliquot of the extract was mixed with 3 mL of the DPPH solution (62 μg/mL in ethanol). After 40 min of incubation in the dark at room temperature, absorbance (D) was measured at 517 nm using the SF-56 spectrophotometer (Lomo, St. Petersburg, Russia). A DPPH solution was used as a control sample.

Free-radical-scavenging activity was calculated as a percentage of inhibition via the following formula:

X% = (D_control_ − D_sample_/D_control_) × 100,

where D_control_ is the optical density of the control solution containing all reagents except the tested extract, and D_sample_ is the optical density of the sample.

The data obtained were analyzed using a non-linear regression curve on GraphPad Prism 5.0. The results are expressed in terms of DPPH IC_50_, and the concentration of the antioxidant required to cause a 50% loss of DPPH in the DPPH radical-scavenging activity assay was ascertained. Solutions of 6-hydroxy-2,5,7,8-tetramethylchroman-2-carboxylic acid [Trolox] and ascorbic acid (concentrations of 2.5–50.0 μg/mL) served as a positive control. A more detailed description of the method is given in [53].

### 4.5. LC-HRMS Analysis of Phenolics

#### 4.5.1. LC-HRMS

LC-HRMS was conducted at the Core Facility of Mass-Spectrometric Analysis at the Institute of Chemical Biology and Fundamental Medicine SB RAS (Novosibirsk, Russia). An Ultimate 3000 liquid chromatograph (Thermo Fisher Scientific, San Jose, CA, USA) coupled with a Q Exactive HF mass spectrometer (Thermo Fisher Scientific, San Jose, CA, USA) was utilized to determine the phenolic compounds in the hydro-ethanol leaf extracts of *E. sibirica* and *E. tanhoensis* as described in [49]. Briefly, separation was carried out on a Hypersil GOLD aQ reversed-phase column (100 × 2.1 mm, 1.9 μm; Thermo Fisher Scientific) maintained at 40 °C, with a flow rate of 0.4 mL/min. The mobile phases consisted of water with 0.1% formic acid (A) and acetonitrile with 0.1% formic acid (B). The gradient was programmed from 5% to 70% B over 40 min, increased to 90% B over 8 min, and returned to 5% B within 5 min. The column was re-equilibrated under the initial conditions for 7 min. The injection volume was 30 μL.

The electrospray ionization source was operated at 3.2 kV in negative-ion mode and 4.2 kV in positive-ion mode, with a capillary temperature of 350 °C and an S-lens RF level of 50. Full-scan MS spectra were acquired in both positive and negative ionization modes over an m/z range of 100–1000 at a resolving power of 120,000 FWHM at *m*/*z* 200, with an AGC target of 1 × 10^6^ and a maximum injection time of 100 ms. Compound identification was performed using full-scan, data-dependent MS/MS (FS-dd-MS^2^). MS/MS spectra were acquired at a resolving power of 30,000 FWHM at *m*/*z* 200 using a 1.0 *m*/*z* isolation window and stepped normalized collision energies of 20, 40, and 60, with an AGC target of 1 × 10^5^, a maximum injection time of 100 ms, and a loop count of 10.

#### 4.5.2. Compound Annotation

The LC-HRMS data were analyzed in Xcalibur 4.2 and Compound Discoverer 3.2 software (Thermo Fisher Scientific, San Jose, CA, USA). All the samples, including blank samples, were assayed in triplicate. All the samples were processed in Compound Discoverer 3.2 via a common workflow called “Environmental Unknown ID w Online and Local Database Searches.” A mass tolerance of 10 ppm was applied during accurate-mass-based processing, elemental composition prediction, and exact-mass database searches. Compound annotation was subsequently performed using complementary approaches based on accurate mass, MS/MS spectral matching, and, where available, reference standards. A more detailed procedure for substance annotation is described in ref. [15]. Several databases, i.e., KEGG (Available online: https://www.genome.jp/kegg/ (accessed on 5 August 2026)), MassBank (Available online: https://massbank.eu/MassBank/ (accessed 5 August 20264)), PlantCyc (Available online: https://plantcyc.org/ (accessed 1 August 2026)), Planta Piloto de Quimica Fina Universidad de Alcala (Available online: http://www.cqab.eu/index.php/en/ (accessed 1 June 2026)), AraCyc (Available online: https://plantcyc.org/database/15-0/ (accessed 1 June 2026)), Extrasynthese (Available online: https://www.extrasynthese.com/ (accessed 1 June 2026)), Indofine (Available online: https://www.indofinechemical.com/ (accessed 1 June 2026)) and Sequoia Research Products (Available online: http://www.chemcd.com/supplier/sequoia.html (accessed 1 June 2026)) were chosen in ChemSpider.

Several mzVault libraries were used in the mzVault Search node: Creation of a PlantMetabolite Spectral Library for Untargeted and targetedMetabolomics.db [55], Negative ionmode_Jan2021.db, Positive ion mode_Jan2021.db (Available online: https://more.bham.ac.uk/bamcg/uhplc-retention-time-and-ms-ms-mass-spectral-fragmentation-libraries/ (accessed 1 June 2026)) and MS2_library.db (Available online: https://doi.org/10.1016/j.jchromb.2020.122105 (accessed 1 June 2026)). Metabolites were annotated on the basis of both accurate mass and fragment mass “fingerprint” spectra via searches against the spectra of compounds available in the mzCloud database (https://www.mzcloud.org; last accessed 1 June 2026) and mzVault. Compound Discoverer spectral-match scores were considered supporting evidence for compound annotation together with accurate-mass information. Compounds confirmed by analysis of authentic reference standards under the same experimental conditions were assigned MSI Level 1. Compounds without authentic standards but supported by matching experimental and library MS/MS spectra were considered putatively annotated compounds (MSI Level 2). When no suitable mzCloud or mzVault spectral match was available, putative annotations based on accurate mass were obtained by searches in ChemSpider and reported as exact-mass-based annotations (MSI Level 3).

According to the workflow, the masses extracted from the chromatograms were aligned and filtered to remove (i) background compounds present in the blank sample and (ii) features without a database-supported annotation.

### 4.6. Statistical Processing of the Results

The data were obtained from at least three independent biological replicates. Leaf samples from each population were randomly divided into three portions. Leaf samples from each portion were then analyzed. The data were analyzed using Microsoft Excel 2010, Statistica 8, and GraphPad Prism v. 5.0. The data were presented as mean ± standard deviation. The statistical significance of the difference in substance levels between *Eranthis* species extracts was assessed using Student’s *t*-test. DPPH radical-scavenging data were subjected to analysis of variance (ANOVA) followed by post hoc comparisons (Tukey’s test; *p* ≤ 0.05). The obtained data meet the mathematical requirements of the statistical analyses used.

## 5. Conclusions

This study expands the phytochemical characterization of the Southeastern Siberian endemics *Eranthis sibirica* and *E. tanhoensis* through comparative LC-HRMS phenolic profiling and DPPH radical-scavenging data. The leaf extract of *E. tanhoensis* proved to be a richer source of phenolic compounds than that of *E. sibirica*. Furthermore, the *E. tanhoensis* extract exhibited higher antioxidant activity compared to the *E. sibirica* extract. The observed species differences were obtained from the specific sampled populations and require validation across independent populations.

Further research is necessary to identify the most active compounds responsible for the antioxidant properties of these plant extracts.

## Figures and Tables

**Table 1:** Antioxidant activity and phenolic compound content in the leaf extracts of *E. tanhoensis* and *E. sibirica*.

Biologically Active Compounds	Contents of Phenolic Compound Classes (g/100 g Dry Weight)	
	*E. tanhoensis*	*E. sibirica*
Phenolic compounds (Gallic acid equivalent)	3.07 ± 0.03 *	2.25 ± 0.03
Tannins (Tannin equivalent)	12.27 ± 0.23 *	10.05 ± 0.19
Catechins (Catechin equivalent)	0.06 ± 0.00 *	0.02 ± 0.00
Flavonoids (Rutin equivalent)	3.88 ± 0.03 *	1.35 ± 0.01
Phenolic acids (Caffeic acid equivalent)	4.50 ± 0.18 *	2.87 ± 0.11
**Antioxidant activity**		
**IC_50_** **(DPPH, µg/mL dry weight)**	*E. tanhoensis*	268.45 ± 8.62 ^c^
	*E. sibirica*	416.0 ± 10.41 ^d^
	Trolox	7.74 ± 0.29 ^a^
	Ascorbic acid	8.69 ± 0.25 ^b^

The table shows the mean ± standard deviation of three indicators, *n* = 3. IC_50_ is the extract/antioxidant concentration that yields 50% inhibition of the 1,1-diphenyl-2-picrylhydrazyl (DPPH) radical. Statistically significant differences are marked with an asterisk (*) (Student’s *t*-test at *p* < 0.05). Means with different superscript letters (a–d) in each row are significantly different (*p* ≤ 0.05) according to Tukey’s honestly significant difference (HSD) test.

**Table 2:** Flavonoids and phenolic acids confirmed or annotated by LC-HRMS in ethanol leaf extracts of *E. tanhoensis* and *E. sibirica* using mzCloud, mzVault, and ChemSpider databases.

ID	Annotated/Identified Compounds	tR (min)	Calcd. Mass (Da)	Found Mass (Da)	Delta of Mass (ppm)	mzCloud or mzVault Score	ESI Mode	Level of Confidence	Presence of Compounds		References
									*E. tanhoensis*	*E.* *sibirica*	
		Flavonoids									
1	Quercetin *	13.32	302.043	302.043	0.09	98.0 ^1^	p	1	+	+	[13,14,15]
2	Quercetin-3β-D-glucoside	13.33	464.096	464.094	−2.23	88.1 ^1^	p	2	+	+	[14,15]
3	Hyperoside *	11.93	464.096	464.095	−0.3	92.7 ^2^	p	1	+	+	[14,15]
4	Reynoutrin	14.44	434.085	434.083	−3.56	88.0 ^1^	p	2	+	+	[14,15]
5	2-(3,4-dihydroxyphenyl)-5,7-dihydroxy-3-{[(2S,3R,4S,5S)-3,4,5-trihydroxyoxan-2-yl]oxy}-4H-chromen-4-one	11.94	434.085	434.085	−0.53	87.6 ^1^	p	2	+	+	-
6	Astilbin	15.84	450.116	450.114	−4.97	66.0 ^1^	n	2	+	+	-
7	Quercetin-3-O-arabinosyl-diglucoside	1.93	758.191	758.190	−0.75	83.4 ^1^	p	2	+	+	-
8	Quercetin 3-O-sambubioside	11.94	596.138	596.137	−1.29	-	p	3	+	+	[14,15]
9	Quercetin-6-O-β-D-xylopyranosyl-β-D-glucopyranoside	11.91	596.138	596.135	−5.09	86.0 ^1^	n	2	+	+	[14,15]
10	Rutin *	12.18	610.153	610.151	−4.19	95.6 ^1^	n	1	+	+	-
11	Kaempferol *	12.84	286.048	286.048	0.23	97.2 ^1^	p	1	+	+	[12,14,15]
12	Kaempferol-7-glucoside	14.43	448.101	448.098	−4.98	76.2 ^1^	n	2	+	+	-
13	Trifolin	12.85	448.101	448.100	−0.43	88.3 ^1^	p	2	+	+	[14,15]
14	Juglalin	15.59	418.090	418.088	−4.91	84.0 ^1^	n	2	+	+	[14,15]
15	Kaempferol-3-O-arabinopyranosyl-glucopyranoside	12.82	580.143	580.141	−3.75	89.3 ^1^	p	2	+	+	-
16	Aromadendrin	21.79	288.063	288.063	−2.02	-	p	3	+	+	[12,14]
17	3,5,7-trihydroxy-2-(3-hydroxy-4- {[(2S,3R,4S,5S,6R)-3,4,5-trihydroxy-6- (hydroxymethyl)oxan-2-yl]oxy}phenyl)- 4H-chromen-4-one	12.71	464.095	464.096	0.89	88.8 ^1^	p	2	+	+	-
18	4-Methoxyphlorizin	13.21	450.153	450.151	−3.57	67.8 ^1^	p	2	+	+	-
19	Aspalathin	13.03	452.132	452.132	−0.22	-	p	3	+	+	[12,14]
20	Isoliquiritigenin	16.76	256.074	256.073	−2.79	71.6 ^1^	p	2	+	+	-
21	Phloretin	14.62	274.084	274.083	−3.85	58.3 ^1^	p	2	+	+	[14,15]
22	Phloridzin	18.10	436.137	436.137	−0.24	-	p	3	+	+	[12,14,15]
23	Auriculoside	13.68	450.153	450.150	−5.97	-	p	3	+	+	[12,14]
24	Chrysin	27.22	254.058	254.057	−3.55	77.0 ^1^	p	2	+	+	-
25	Norwogonin	20.32	270.053	270.049	−13.19	71.2 ^1^	p	2	+	+	-
26	Orientin *	14.44	448.101	448.098	−5.93	-	p	1	+	+	[13,14,15]
27	Carlinoside	12.37	580.143	580.140	−4.16	-	p	3	+	+	[13,14,15]
28	Genistein	15.98	270.053	270.053	0.70	76.2 ^1^	p	2	+	+	-
29	Catechin (cianidanol)	14.79	290.079	290.079	−0.88	61.2 ^1^	p	2	+	+	[14,15]
30	Naringenin	29.19	272.068	272.068	−1.94	54.8 ^2^	p	2	+	+	-
31	5,7,4’-Trihydroxy-6-methylflavanone	27.72	286.084	286.083	−3.25	67.1	p	2	+	+	-
32	Pinocembrin	28.18	256.074	256.073	−2.04	76.6 ^2^	p	2	+	+	-
33	Pinostrobin	34.48	270.089	270.088	−3.36	85.1 ^1^	p	2	+	+	-
34	Prunin	14.50	434.121	434.120	−4	54.4 ^1^	p	2	+	+	-
35	2-(3,4-Dihydroxyphenyl)-5,7-dihydroxy- 4-oxo-4H-chromen-3-yl 6-O-(6- deoxyhexopyranosyl)-4-O-[(2E)-3-(4- hydroxyphenyl)-2- propenoyl]hexopyranoside	15.87	756.190	756.187	−3.80	76.8 ^1^	n	2	+	+	-
36	(2S,3R)-3,5-Dihydroxy-2-(4-hydroxyphenyl)-3,4-dihydro-2H-chromen-7-yl β-D-glucopyranoside	16.77	436.137	436.134	−6.41	65.8 ^1^	p	2	+	+	-
		**Phenolic acids and derivatives**									
37	Benzoic acid *	10.14	122.037	122.035	−12.92	76.7 ^1^	n	1	+	+	-
38	Gentisic acid *	27.58	154.027	154.027	0.17	-	p	1	+	+	[13]
39	4-(Octyloxy)benzoic acid	41.64	250.157	250.155	−5.81	69.3 ^1^	n	2	+	+	-
40	4-Isobutylbenzoic acid	14.91	178.099	178.099	−2.97	63.6 ^1^	p	2	+	+	-
41	Methyl salicylate	16.40	152.047	152.046	−10.71	76.3 ^1^	n	2	+	+	-
42	2-Hydroxycinnamic acid	18.00	164.047	164.047	−3.82	76.4 ^1^	p	2	+	+	-
43	Caffeic acid *	10.02	180.042	180.041	−9.38	-	n	1	+	+	[13]
44	1,6-Bis-O-[(2E)-3-(4-hydroxyphenyl)-2-propenoyl]-β-D-glucopyranose	18.00	472.137	472.135	−5.15	78.41 ^1^	n	2	+	+	-

* The compound was confirmed by means of a reference standard; Score databases: ^1^ mzCloud; ^2^ mzVault; “-” ChemSpider only; tR—retention time; Mode: p—positive ESI; n—negative ESI; References—literature sources where compounds have previously been reported for plant species of the genus *Eranthis*; confidence level: 1—metabolites identified using standards (MSI level 1), 2—compounds putatively annotated matching experimental MS/MS spectra against library spectra in mzCloud or mzVault (MSI Level 2), 3—compounds tentatively annotated using a ChemSpider search (MSI level 3).

**Table 3:** DPPH radical-scavenging activities of some phenolic compounds (literature data).

Phenolics	Molecular Formula	Free OH	Antioxidant Activity (DPPH) IC_50_ (μg/mL)	References
Quercetin *	C_15_H_10_O_7_	3,5,7,3’,4’	1.8–9.0	[31,32,33,34,35]
Quercetin-3β-D-glucoside	C_21_H_20_O_12_	5,7,3’,4’ **	7.1–12.0	[32,34]
Hyperoside	C_21_H_20_O_12_	5,7,3’,4’ **	5.4	[32]
Reynoutrin	C_20_H_18_O_11_	5,7,3’,4’ **	0.25	[36]
Kaempferol	C_15_H_10_O_6_	3,5,7,4’	5.3–13.2	[31,32,33,35]
Rutin	C_27_H_30_O1_6_	5,7,3’,4’ **	4.9–16.0	[31,34]
Astilbin	C_21_H_22_O_11_	5,7,3’,4’ **	7.3	[37]
Kaempferol-7-glucoside	C_21_H_20_O_11_	3,5,4’ **	51.1	[38]
Trifolin	C_21_H_20_O_11_	3,5,4’ **	77.0	[39]
Juglalin	C_20_H_18_O_10_	5,7,4’ **	>100.0	[31]
Phloretin	C_15_H_14_O_5_	2,4,4’,6’	48.6	[40]
Phloridzin	C_21_H_24_O_10_	2,4,4’ **	>500.0	[41]
Chrysin	C_15_H_10_O_4_	5,7	>100.0	[31,33]
Norwogonin	C_15_H_10_O_5_	5,7,8	35.6	[33]
Orientin	C_21_H_20_O_11_	5,7,3’,4’ **	>100.0	[42]
Genistein	C_15_H_10_O_5_	5,7,4’	>100.0	[31]
Catechin	C_15_H_14_O_6_	3,5,7,3’,4’	14.3	[33]
Naringenin	C_15_H_12_O_5_	5,7,4’	>100.0	[31]
Pinostrobin	C_16_H_14_O_4_	5	62.8	[43]
Caffeic acid	C_9_H_8_O_4_	3,4 **	4.5	[32]

* Cross-study comparisons are qualitative only; ** phenolic OH only. IC_50_ values come from various studies under different assay conditions.

## Data Availability

The data presented in this study are available on request from the corresponding author.

## Supplementary Materials

The following supporting information can be downloaded at https://www.mdpi.com/article/10.3390/ijms27219973/s1.
