# Supplementary material for: Phenolic Profiles and DPPH Radical-Scavenging Activity of Two Eranthis Species from Southeastern Siberia
Source: Int J Mol Sci. 2026 Nov 6;27(21):9973. doi: 10.3390/ijms27219973 (PMC13763778; doi:10.3390/ijms27219973)
Supplement: Supplementary file 1 [file ijms-27-09973-s001.zip › ijms-4608840-supplementary.pdf]

Supplementary Materials

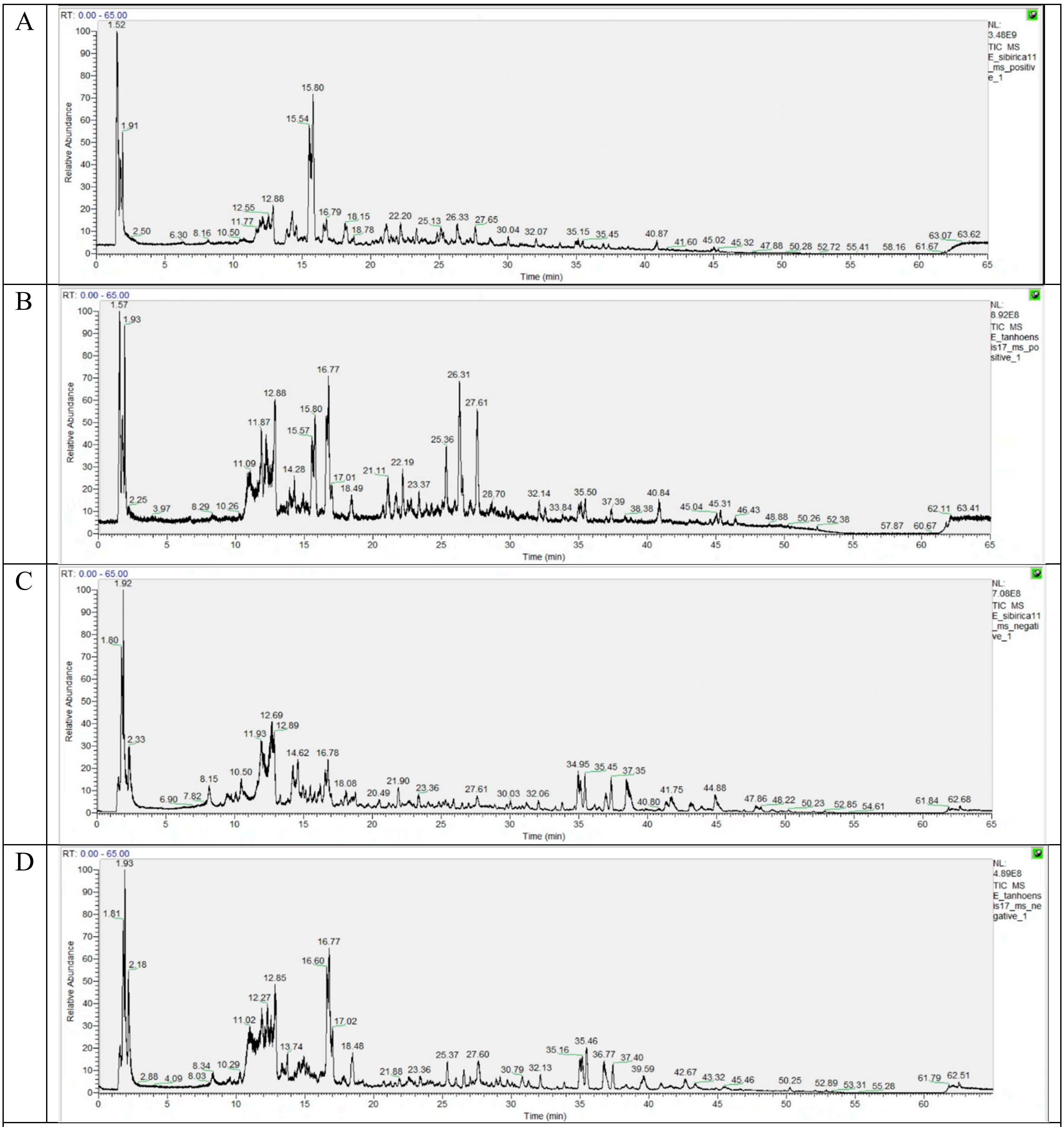

Figure S1. Total ion current (TIC) chromatograms of *E. sibirica* (A, C) and *E. tanhoensis* (B, D) in positive (A, B) and negative (C, D) ion modes.
